# Harnessing human plasmacytoid dendritic cells as professional APCs

**DOI:** 10.1007/s00262-012-1210-z

**Published:** 2012-02-01

**Authors:** Jurjen Tel, Anne M. van der Leun, Carl G. Figdor, Ruurd Torensma, I. Jolanda M. de Vries

**Affiliations:** 1 Department of Tumor Immunology, Radboud University Nijmegen Medical Centre and Nijmegen Centre for Molecular Life Sciences, PO Box 9101, 6500 HB Nijmegen, The Netherlands; 2 Medical Oncology, Radboud University Nijmegen Medical Centre and Nijmegen Centre for Molecular Life Sciences, Nijmegen, The Netherlands

**Keywords:** PIVAC 11, Plasmacytoid dendritic cells, Antigen-uptake receptors, Antigen presentation, Targeting

## Abstract

The plasmacytoid dendritic cell (pDC) constitutes a unique DC subset that links the innate and adaptive arm of the immune system. Whereas the unique capability of pDCs to produce large amounts of type I IFNs in response to pathogen recognition is generally accepted, their antigen-presenting function is often neglected since most studies on antigen presentation are aimed at other DC subsets. Recently, pDCs were demonstrated capable to present antigen leading to protective tumor immunity. In this review, we discuss how pDCs could be exploited in the fight against cancer by analyzing their capacity to capture, process and (cross-) present antigen.

## Introduction

The human immune system constitutes of a wide variety of cell types to maintain immune homeostasis. In this system, professional antigen-presenting cells (APCs) control the tight balance between tolerance and immunity. All professional APCs exploit an efficient antigen-uptake machinery and long-lived MHC class II-peptide complexes on the cell surface. Dendritic cells (DCs) represent a family of professional APCs that are derived from hematopoietic precursors and have the capacity to induce antigen-specific T-cell responses. Efficient priming is dependent on full maturation of DCs, which is evoked by the recognition of specific pathogen-associated molecular patterns by their distinct pathogen recognition receptors (PPRs). Some of the best characterized PPRs expressed by DCs are the toll-like receptors (TLRs) that are able to bind different pathogen structures like LPS, lipoprotein structures, DNA or single- and double-stranded RNA motifs, thereby initiating a signaling cascade leading to upregulated expression of MHC and co-stimulatory molecules on the DC surface, presentation of antigens and enhanced cytokine production [1, 2]. After infection or inflammation, this maturation process enables DCs to migrate to the lymph nodes and presents encountered antigens to naïve T cells.

The DC family is very heterogeneous and consists of different DC subsets each with specific functional characteristics. In general, two different DC subtypes can be distinguished, for example, myeloid DCs (mDCs) and plasmacytoid DCs (pDCs). These distinct subsets express various surface receptors and PPRs, which determine their specialized functions (Table 1) [3]. The mDC subset can be identified and subdivided in three different subtypes by the expression of CD11c in combination with their unique surface molecules CD1c (BDCA1), CD141 (BDCA3) and CD16 [4]. Classically, mDCs reside in peripheral tissues in an immature state and migrate to lymph nodes after their maturation where they produce IL-12 and activate T cells [5, 6]. pDCs are an extraordinary subset that differs from the other DC subsets by a specific feature: the capacity to produce large amounts of type I IFNs in response to viral or bacterial stimuli. pDCs employ TLR7 and TLR9, located in intracellular endosomes and lysosomes, to recognize single-stranded viral RNA or unmethylated CpG DNA motifs, respectively [7, 8]. Through the secretion of high levels of type I IFNs, pDCs communicate with other immune cells, for example, they stimulate mDCs to enhance T-cell activation and activate natural killer cells and B cells. In this way, pDCs link the innate and adaptive arm of the immune response [2, 9]. In contrast to mDCs, pDCs are hardly found in peripheral tissues under steady-state conditions. Instead, pDCs circulate through the body after entering the bloodstream and reach secondary lymphoid organs via high endothelial venules, while most other DC subsets enter secondary lymphoid organs via the lymph vessels. Following inflammation, pDCs leave the bloodstream and accumulate in the infectious site to take up antigens, followed by migration to lymph nodes to present the encountered antigens [10]. The unique capability of pDCs to produce large amounts of type I IFNs in response to pathogen recognition is well accepted. However, the position of pDCs as professional APCs has long been dictated by the view that pDCs are inferior to mDCs as it comes to antigen presentation. In this review, we discuss how pDCs could be exploited in the fight against cancer by analyzing their capacity to capture, process and (cross-) present antigen.

**Table 1:** Phenotypical and functional characteristics of blood DC subsets [3, 6, 45, 62, 82–84]

		pDCs		mDCs					
				BDCA-1		BDCA-3		CD16	
		Steady state	Activated	Steady state	Activated	Steady state	Activated	Steady state	Activated
Phenotype	CD4	++	++	+	++	+	++	+	++
	CD11c	−	−	+++	+++	++	+++	+++	+++
	CD40	+/−	+++	+/−	+++	+/−	+++	+/−	+++
	CD80	−	++	−	++	−	++	−	++
	CD83	−	+	−	+	−	+	−	+
	CD86	+	+++	+	+++	+	+++	++	+++
	HLA-DR	++	+++	++	+++	++	+++	++	+++
	HLA-ABC	+/−	++	+	++	+	++	+	++
	CCR7	−	++	−	++	−	++	−	++
Toll-like receptors	TLR-1	+		+		+		+	
	TLR-2	−		++		++		++	
	TLR-3	−		++		++		−	
	TLR-4	−		+		+		+	
	TLR-5	−		+		+/−		+	
	TLR-6	+/−		+		+		+	
	TLR-7	++		+/−		+/−		+/−	
	TLR-8	–		+		+		+	
	TLR-9	+++		−		−		−	
	TLR-10	+		+		+		+	
C-type lectin receptors	DEC-205	++		++		+++		+	
	DCIR	+		+		−		+	
	BDCA-2	++		−		−		−	
	CLEC9a	−		−		+		−	
Fc receptors	FcαR	+/−		−		−		−	
	FcεRI	+		+		−		−	
	FcγRI	−		−		−		+	
	FcγRIIa	+		+		+		+	
	FcγRIIb	−		+		+		+	
	FcγRIII	−		+		−		+/−	
Upon activation									
Cytokine secretion	IFNα	+++		−		−		−	
	IFNβ	+++		+		+		+	
	IFNω	++		−		−		−	
	IFNλ	+		+		+		+	
	IL-1β	+		+		+		+	
	IL-6	++		++		++		+++	
	IL-8	+++		+++		+++		+++	
	IL-12	−		+		+		+	
	TNFα	+++		+++		+++		+++	
Migration		+		+		+		+	
Ag (cross-) presentation	CD4	+		+		+		+	
	Th1^a^	+		+		+		+	
	CD8	+		+		+		+/−	

^a^After activation, pDC-derived type I IFNs and mDC-derived IL-12 are involved in the differentiation of Th1 cells

## pDCs induce antitumor immune responses leading to protective immunity

Most DC-based immunotherapy has been performed with monocyte-derived DCs that were generated ex vivo [11–13]. Although clinical outcomes were observed in a fraction of patients treated with DCs [12–17], it has been postulated that moDCs might be less effective than naturally occurring DC subsets. pDCs comprise one of these natural DC subsets circulating in the blood and are potential candidates for DC-based antitumor immunotherapy as detailed below.

The observation that human pDCs were able to induce T-cell responses in vitro confirmed results observed in mice where CpG or influenza virus matured pDCs-induced ovalbumin (OVA)-specific CD4^+^ and CD8^+^ T-cell responses [18–22]. While CpG-matured pDCs induced CD8^+^ T-cell responses against endogenous antigens, no T-cell responses were observed against exogenous OVA antigen [23]. This discrepancy could in part be attributed to the fact that in some studies, pDCs were pulsed with the entire OVA protein, while others used OVA fragments. In other studies with exogenous antigens like influenza A or HSV, pDCs also induced CD4^+^ and CD8^+^ T-cell responses, again indicating their capacity to present antigens and stimulate T cells [24–26]. Notwithstanding these results, pDCs were less effective in CD4^+^ priming when compared to mDCs when both are stimulated with TLR agonists, like LPS [19]. This might be explained by the fact that mDCs express high levels of TLR4 where pDCs do not. Salio et al. [23] proposed that high numbers of peptide-pulsed pDCs are equally efficient as peptide-pulsed mDCs in priming of CD8^+^ T cells. This notion is supported by the finding that murine pDCs exposed to influenza virus induced the generation of memory CD8^+^ T cells as well as effector CD8^+^ T cells upon rechallenge with the virus, leading to protective immunity [20]. Further evidence that pDCs can induce protective immunity comes from studies where mice were vaccinated with CpG-matured tumor peptide-pulsed pDCs or pulsed by a *Leishmania major* (*L. major*) lysate. Mice vaccinated with tumor peptide-loaded pDCs gained antitumor immunity and were protected upon tumor challenge, whereas nonvaccinated animals showed unhampered tumor growth [23]. This indicates that mature pDCs can effectively generate protective immunity [27]. Together, these data emphasize the potential of pDCs as type I IFN-secreting professional APCs.

## Antigen cross-presentation by pDCs

**Fig. 1:**
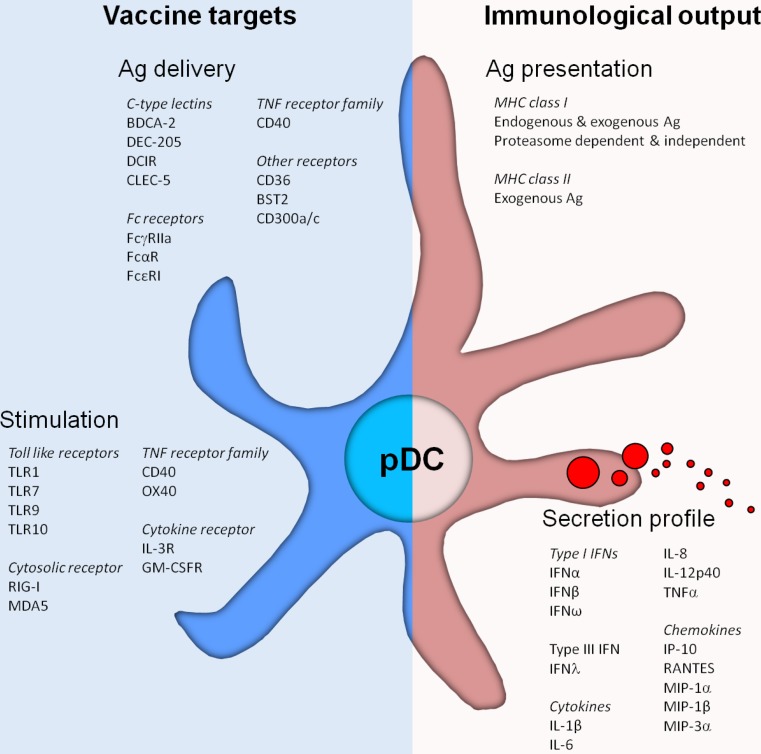
Plasmacytoid dendritic cells (pDCs) express a wide variety of pattern recognition receptors involved in pDC function. These receptors can be harnessed to facilitate the targeted delivery of antigen to pDCs, leading to antigen (cross-) presentation and activation of both CD4^+^ and CD8^+^ T cells. Furthermore, receptors that are involved in the recognition of pathogen-associated molecular patterns induce the activation of human pDCs leading to phenotypical maturation as well as the secretion of cytokines and chemokines. Those soluble factors on their turn can attract and induce the activation of other immune cells, thereby enhancing the immune response. The combination of “vaccine targets” for antigen delivery and stimulation largely affects the “immunological output” generated by pDCs

A potent immune response requires both CD4^+^ T-cell and effector CD8^+^ responses. To this end, the capacity of DCs to cross-present antigen is absolutely essential. In contrast to the well-defined capacity of human CD141^+^ mDCs and mouse CD8α^+^ mDCs to cross-present antigens [28–31], controversy exists about ability of pDCs to cross-present. Although in some studies murine pDCs fail to induce functional CD8^+^ T cells [18, 32], human pDCs appear to be competent in processing exogenous antigens to be presented in MHC class I molecules [33–35] (Fig. 1). Exogenous antigens captured by pDCs might end up in MHC class I through distinct routes; via an endosome-to-cytosol pathway or via a cytosol-independent pathway [36–38]. Di Pucchio et al. [35] showed that a functional proteasome is not necessary for pDCs to present captured viral antigens in MHC class I molecules, indicating that pDCs have the capacity to process and load captured antigens directly onto MHC class I molecules in endosomal compartments. This is in contrast with findings showing that exogenous antigens need to be transported from endosomal vesicles into the cytosol, become processed by the proteasome and subsequently loaded on MHC class I molecules, similar to endogenous antigens [39, 40]. Other studies showed that pDCs can cross-present antigens directly after stimulation with viral pathogens with comparable efficacy as mDC subsets. It has been reported that pDCs have larger amounts of MHC class I molecules stored in endosomes when compared to other DC subsets and can therefore rapidly transport these MHC class I molecules to the plasma membrane [35]. Taken together, studies performed with human pDCs suggest that an effective cross-presentation machinery is present. Human pDCs can be regarded as professional APCs and should therefore be considered for immunotherapy.

## Antigen uptake by pDC

Two main strategies are exploited in DC-based cancer immunotherapy: (1) isolation and ex vivo stimulation of autologous DCs and (2) via direct targeting of DCs in vivo. Until now, clinical trials have primarily focused on the generation, stimulation and manipulation of DCs ex vivo*.* To compose a potent vaccine, the antigenic cargo needs to be efficiently and specifically delivered, avoiding inappropriate release of vaccine content. Furthermore, to explore the most potent mechanism to stimulate immature pDCs with antigens in vivo, it is important to dissect the antigen-uptake and stimulation mechanisms of pDCs. Immature DCs are able to take up various types of exogenous antigens through macropinocytosis, phagocytosis or receptor-mediated endocytosis [41]. Macropinocytosis refers to a process that mediates nonspecific capture of soluble antigens, without the involvement of receptors. While phagocytosis comprises receptor-mediated engulfment of larger antigenic particles or pathogens, receptor-mediated endocytosis is considered the most specific and efficient mechanism to capture antigens [41].

### Phagocytosis and pinocytosis

Like reported for immature mDCs, immature pDCs can phagocytose viral pathogens such as cytomegalovirus, HIV-I and influenza A resulting in priming naïve CD8^+^ T cells [42, 43]. In contrast to their ability to engulf viruses, there has been some controversy whether or not pDCs are involved in the uptake of bacteria. Some studies demonstrate that pDCs were able to take up and get activated by bacteria like S. pyogenes and S. aureus [43, 44], while Piccioli et al*.* [45] showed that pDCs were not able to phagocytose nor get activated by S. aureus. Other studies demonstrated that pDCs hardly take up apoptotic cells compared with mDCs [46, 47], while Hoeffel et al. [39] showed that pDCs are capable of taking up apoptotic cell fragments. These opposing results suggest that pDCs are endowed with specific PRRs that are involved in the specific recognition of particular bacteria [43]. Other studies have focused on the mechanisms for taking up soluble antigens including KLH and OVA. Immature pDCs are likely capable of taking up soluble antigens, but less efficient than mDCs [18, 21, 48]. This difference in effectiveness could be caused by the lower macropinocytosis activity of pDCs compared with mDCs [49].

Although pDCs are considered to be ineffective in engulfing particulate antigens like bacteria and apoptotic cells, we previously demonstrated that human pDCs induced immune responses after internalizing particulate matter [50]. Stimulating pDCs with polylactic-co-glycolic acid (PLGA) microparticles packed with antigens and TLR agonists resulted in the activation of antigen-specific CD4^+^ T cells [50]. Thus, although pDCs are less efficient than mDCs, they can phagocytose and process particles, which simultaneously release TLR agonists and antigens. As a consequence, pDCs mature and MHC class II molecules are loaded with antigens. Interestingly, although human pDCs were found to be less efficient when compared to mDCs in taking up particulate compounds, both cell types were fully matured. These findings suggest that pDCs might even be more competent in handling engulfed materials than mDCs.

### Receptor-mediated endocytosis

Next to the ability to take up encountered pathogens and antigens by phagocytosis, pDCs are also known to express a broad repertoire of antigen-uptake receptors on their cell surface (Table 1; Fig. 1). This large repertoire facilitates the uptake of encountered pathogens and antigens via receptor-mediated endocytosis, resulting in more efficient antigen uptake than via phagocytosis.

#### Fc receptors

One of the best defined receptor families relevant for the uptake of microbial pathogens is the Fc receptor family. They mediate endocytosis and presentation of antigens by capturing immune complexes. Fc receptors do not mediate uptake of soluble antigens, but antigens forming immune complexes with antibodies [51, 52]. The human FcγR family consists of the activating receptors FcγRI (CD64), FcγRIIa/c (CD32a/c) and FcγRIIIa/b(CD16), and the inhibitory receptor FcγRIIb (CD32b) [53]. pDCs express FcγRIIa [54, 55], FcαR (unpublished observation) and FcєRI [56], but not FcγRI and FcγRIIIa/b (Table 1). FcγRIIa-mediated internalization stimulates the redistribution of MHC class II molecules from lysosomal vesicles to the plasma membrane, facilitating presentation of antigen-IgG complexes captured by FcγRIIa in both MHC class I and MHC class II molecules [54, 57]. The FcγRIIa surface expression levels are similar for immature and mature pDCs. However, triggering of TLRs results in decreased endocytosis via FcγRIIa, indicating impaired internalization after pDC maturation. This might prevent the uptake of antigens, which are not associated with TLR activation, to be captured after maturation [55]. Whether FcαR and FcєRI expressed by human pDCs are involved in antigen uptake and presentation remains to be established. Triggering FcєRI or FcγRIIa does not affect the expression of surface receptors involved in providing co-stimulatory signals during T-cell activation [54]. However, these receptors differ in their ability to modulate the secretion of type I IFN by pDCs in response to TLR ligation. Although both receptors employ an ITAM for downstream signaling, FcєRI triggering strongly impairs TLR9-induced IFNα secretion [56], while FcγRIIa triggering leaves TLR9-induced IFNα secretion unaffected [55].

#### C-type lectin receptors on pDCs

Another endocytic receptor family expressed on the immature pDCs are the C-type lectin receptors (CLRs) (Fig. 1). CLRs recognize carbohydrate moieties leading to internalization, the “C” indicating Ca^2+^ dependence of carbohydrate binding [58]. BDCA-2 is a type II CLR exclusively expressed by immature pDCs and used as a marker to distinguish immature pDCs from mDCs and other immune cells [59]. BDCA-2 is shown to capture antigens decorated with carbohydrates, leading to presentation of the captured antigen on MHC class II molecules. However, triggering BDCA-2 impairs TLR9-mediated type I IFN secretion by pDCs, thereby attenuating the induction of innate immune responses [60]. Furthermore, BDCA-2 expression is reduced by TLR7 and TLR9 signaling, indicating that the endocytic function of BDCA-2 is primarily important in immature pDCs [61]. In addition to BDCA-2, pDCs also express the more broadly expressed CLRs DEC-205 and DCIR [62, 63]. Triggering DEC-205 and DCIR both inhibit TLR9-mediated type I IFN secretion by pDCs, but to a lesser extent than triggering through BDCA-2. Moreover, neither DEC-205 nor DCIR affect the expression of co-stimulatory molecules on the plasma membrane [62, 64]. While the expression and/or scavenging function of most endocytic receptors on pDCs, such as DCIR, is downregulated upon TLR-induced activation, DEC-205 expression is enhanced and still able to capture and internalize antigens [62]. Like antigen capture by other CLRs, uptake of antigens by DEC-205 and DCIR leads to antigen processing and presentation in MHC class II molecules, followed by the induction of CD4^+^ T-cell responses [62, 63]. Interestingly, under same conditions targeting of DEC-205 on pDCs induced highly similar T-cell responses compared to responses induced by CD1c^+^ mDCs and moDCs [62]. These findings reveal that pDCs, although generally considered inferior, are as efficient and professional as other mDCs, generally accepted as professional APC. Whether BDCA-2, DEC-205 and/or DCIR is involved in antigen cross-presentation by human pDCs remains to be elucidated. Initial observations that mDCs can cross-present antigens captured by DEC-205 and DCIR suggest that both receptors might be able to perform a similar role in pDCs as well [63, 65–67].

## Targeted delivery: Which receptor to aim for?

Facilitating the targeted delivery of particulate vaccines to DCs in vivo via receptor-mediated endocytosis seems an interesting opportunity, since this mechanism allows the specific and simultaneous delivery of maturation factors and antigens. But which receptor should be selected to obtain a potent immune response when considering antigen-presenting pDCs?

Several studies identified receptors for in vivo targeting of pDCs. In mice, Siglec-H and bone marrow stromal cell antigen 2 (BST2) were identified as pDC-specific receptors that opt for interesting targets [68]. Siglec-H is an endocytic receptor and member of the Siglec receptor family. Although most Siglec family members contain an ITIM sequence, Siglec-H lacks this domain [69, 70]. Siglec-H associates with DAP-12, which in spite of having this ITAM motif, result in impaired secretion of type I IFN [71, 72]. In mice, Siglec-H is involved in antigen cross-presentation, as demonstrated by the priming of antigen-specific CD8^+^ T cells upon antigen uptake via Siglec-H [69]. Recently, Loschko et al. [73] underscored the potency of targeting pDCs in vivo via BST2. In their study, they reported that targeted pDCs were efficient inducers of the expansion of both antigen-specific CD4^+^ and CD8^+^ T-cell responses. Interestingly, they observed protective antitumor responses when targeting pDCs with simultaneous administration of a TLR agonist [73]. This makes Siglec-H and BST2 potent receptors for targeting murine pDCs for the induction of CD8^+^ T-cell responses and protective tumor immunity. Although specific for murine pDCs, human pDCs do not express Siglec-H [74]. Freshly isolated as well as activated human pDCs do express BST2 [75]. However, in man expression of BST2 is not restricted to pDCs as it is also expressed by B cells. Moreover, it is upregulated on various cells upon IFNα treatment. Therefore, other receptors expressed by pDCs might be better suited [75]. Regarding the characteristics of the different surface receptors expressed by pDCs, specifically targeting antigen to the ITAM-containing FcγRIIa seems to be a promising strategy to induce immunity, since FcγRII is involved in T-cell priming, and moreover triggering FcγRIIa does not negatively affect type I IFN production. One disadvantage of FcγRIIa is that it is not uniquely expressed by pDCs, meaning that pDCs cannot be specifically targeted by triggering FcγRIIa [52]. Similarly, although DEC-205 expression is largely restricted to DCs in mice, it is broadly expressed on different immune cells in man (Table 1) [76]. In this respect, DCIR is expressed by less diverse immune cell types, making this receptor more potent for specific targeting of pDCs. Nevertheless, targeting DCIR might also activate other DC types like mDCs. Combining the stimulation of both DC types unlocks an interesting approach to potentially establish a more potent vaccine, since interaction between pDCs and mDCs has been demonstrated to increase antigen-specific immune responses. Activating pDCs along with mDCs leads also to induction of innate immune responses, likely resulting in an intensified adaptive immune response. pDCs are found to stimulate and enhance the cytokine secretion and cross-presentation of antigens leading to CD8^+^ T-cell priming by mDCs and induction of an antiviral immune response by moDCs [77–79]. In turn, pDCs cocultured with mDCs are capable of inducing an immune response against bacteria where they fail to respond on their own [45]. In mice, an enhanced antitumor response was found when mDCs and pDCs were cocultured during pulsing with tumor antigens [80]. Moreover, pDCs were found to cross-talk indirectly with mDCs, via activation of specific lymphocyte subsets that can interact with, and might thereby stimulate, mDCs [81]. Together, these observations strongly suggest that combining pDC activation with the activation of other DC subsets might be advantageous and result in a more powerful immune response. Triggering DCIR could potentially establish such a synergetic immune response, while triggering pDC-specific receptors, like BDCA-2, initiate a more restricted induced immune response. Moreover, triggering DCIR does not completely inhibit TLR-induced type I IFN secretion by pDC as would be caused by BDCA-2 ligation. We hypothesize that the locally secreted type I IFN is important to establish an effective immune response, since type I IFNs links innate and adaptive immune responses by cross-talk with mDCs, natural killer T cells, natural killer cells and B cells. Alternatively, targeting CD40 could be considered, since it induces TLR-independent pDC maturation without negatively affecting type I IFN secretion. However, like FcγRIIa, BST2 and DEC-205, CD40 is expressed on many different cell types other then DCs and might therefore be less powerful for targeting pDCs.

## Conclusion

Human pDCs seem well equipped for therapeutic strategies aimed at eliciting specific immune response and tumor eradication. Although there is still some controversy on the cross-presenting ability of pDCs, several studies demonstrate that they can cross-present antigens and effectively induce antigen-specific CD8^+^ T-cell responses leading to protective tumor immunity in both mice and man. Therefore, it might be interesting to directly target pDCs in vivo to simultaneously deliver TLR agonists and antigens. Based on existing literature, DCIR seems a potent target, since triggering of this receptor leads to antigen presentation by pDCs as well as other DC subsets without totally blocking the TLR-induced cytokine secretion. Furthermore, combined stimulation of pDCs and mDCs seems to induce a more potent and powerful immune response and therefore deserves more elaborate study. This knowledge will certainly help to map the way for DC-based targeting strategies that can be exploited in autoimmune and infectious diseases as well as in cancer.
